# Nutritional support therapy for liver transplantation in an adult-onset type II citrullinemia patient: a case report

**DOI:** 10.3389/fnut.2024.1364866

**Published:** 2024-03-22

**Authors:** Yao Du, Yang-Yang Fu, Yang Yue, Bing Han, Wen-Jie Zhang, De-Cai Yu, Xiao-Jie Bian

**Affiliations:** ^1^Department of Pharmacy, Nanjing Drum Tower Hospital, Affiliated Hospital of Medical School, Nanjing University, Nanjing, China; ^2^Department of Pharmacy, Nanjing Drum Tower Hospital, School of Basic Medicine and Clinical Pharmacy, China Pharmaceutical University, Nanjing, China; ^3^Department of Hepatobiliary and Transplantation Surgery, Nanjing Drum Tower Hospital, Affiliated Hospital of Medical School, Nanjing University, Nanjing, China

**Keywords:** CTLN2, liver transplantation, nutritional support therapy, MCT, case report

## Abstract

Liver transplantation is an effective measure to treat adult-onset type II citrullinemia (CTLN2). Active and effective perioperative nutrition support is a very important treatment for the prognosis of such patients. In this paper, we analyzed the process, results, and outcome of nutritional support therapy in a case of CTLN2, and concluded that the perioperative nutritional support program for CTLN2 patients should be followed prior to surgery:1.because of the prevalence of severe malnutrition in CTLN2 patients, Enteral nutrition (EN) combined with Parenteral nutrition (PN) should be the first choice for nutritional support; 2. daily energy intake should be 35 ~ 40 kcal/kg; 3. the nutritional formula should be composed of low-carbohydrates and high medium-chain triglyceride (MCT). Postoperative: initiating EN as soon as possible is recommended to restore intestinal function and adjuvant PN might be taken into consideration in the early stage. The purpose of this case was to provide experience for the development and adjustment of the perioperative nutritional support regimen for CTLN2 patients.

## 1 Introduction

Adult-onset type II citrullinemia (CTLN2) is one of the metabolic diseases characterized by hyperammonemia and an elevated level of serum citrulline, which is mainly manifested as wasting and recurring episodes of consciousness disturbance, and shows a dietary preference for protein-and lipid-rich foods (1). It is mostly caused by Citrin deficiency (CD). Citrin is an aspartate-glutamate carrier, which is closely associated with hepatic energy supply. CD is caused by mutations of the *SLC25A13* gene on chromosome 7q21.3 that can cause neonatal intrahepatic cholestasis (NICCD) or CTLN2 (2). Liver transplantation has been considered as the most effective therapy for CTLN2 (3). However, the poor preoperative nutritional status of the patients significantly increases the incidence of post-transplant complications, morbidity and mortality. Therefore, active and efficient nutritional support during the perioperative period is important for the prognosis of patients. Recent studies have indicated that administering arginine and sodium pyruvate alongside a carbohydrate-restricted diet can serve as an effective therapy for patients with CTLN2. Additionally, it has been suggested that providing intensive nutritional support may enhance patient survival (3). The 2020 European Society of Parenteral and Enteral Nutrition (ESPEN) practical guideline on clinical nutrition in liver disease recommends vegetable protein diets as the preferred nitrogen sources for managing hepatic encephalopathy (HE), while suggesting the avoidance of meat proteins as much as possible (4). Previously, various guidelines have emphasized clinical and nutritional aspects for patients with liver diseases such as HE, liver cirrhosis, and liver transplantation. However, limited information is available regarding CTLN2 (4–8). In this case, the patient's serum ammonia levels were progressively elevated despite receiving the regular enteral nutritional (EN) formulations with vegetable protein preoperatively. The clinical pharmacist analyzed the factors that contributed to the patient's systemic condition and worked with the surgeon to adjust the nutrition support regimen, which allowed the patient's serum ammonia to return to normal and improved his nutritional status. The liver transplantation was subsequently carried out successfully. After liver transplantation, the clinical pharmacist performed regular nutritional assessments of the patient and proceeded to alter the nutritional support protocol based on the patient's systemic condition. The report of this case aimed to provide the clinical treatment experience for the development and adjustment of the perioperative nutritional support regimen for CTLN2 patients with liver transplantation.

## 2 Case presentation

A 22-year-old male was admitted to the hospital because of an increased level of serum ammonia for 8 months and an altered sleep pattern lasting over 7 months. He developed aberrant behavior with no apparent cause during the previous 8 months, which presented with blurred consciousness, dressing and undressing without purpose, involuntary vocalizations, accompanied by flapping tremors in both hands, lasting four to 5 h, occurring once every five to 6 days; or presented with nighttime sleep disturbances, irritability, and no response to sound, lasting four to 5 h with relief, occurring once every three to 4 days.

The patient was treated symptomatically at a local hospital, but the same kind of symptoms were recurrent. Then he was transferred to Nanjing Brain Hospital. The cranial magnetic resonance imaging (MRI) revealed signals and perfusion abnormalities as follows: 1. Multiple abnormal signals were detected in the bilateral temporal-parietal cortex, bilateral corona radiata and deep within the left temporal lobes. 2. The bilateral temporal-parietal lobes exhibited hypoperfusion, with regional hyperperfusion noted in the left temporal cortex. Abdominal computed tomography (CT) suggested chronic pancreatitis. For further treatment, he was referred to the department of infectious disease of our hospital. On admission, he weighed 32 kg and was 166 cm tall. His body mass index (BMI) was 11.61 kg/m^2^. The results of laboratory examinations were shown in Table 1. DNA analysis revealed compound heterozygous mutations in *SLC25A13*, leading to the diagnosis of CTLN2. Although the patient underwent conservative therapy with the administration of arginine and ornithine/aspartate to reduce serum ammonia, lactulose for promoting the excretion of intestinal ammonia, diammonium glycyrrhizinate for hepatocyte protection, and ursodeoxycholic acid for jaundice, he still displayed neuropsychiatric symptoms including recurrent episodes of altered consciousness. Therefore, the patient was advised and opted to undergo liver transplantation. After admission, the patient continued to receive conservative therapy. He was alert with able to eat on his own. Results of nutritional risk screening and nutritional assessment were as follows: ① Nutrition Risk Screening 2002 (NRS 2002) score 4 indicated malnutrition risk. ② Patient-Generated Subjective Global Assessment (PG-SGA) score 10 indicated the nutritional intervention was urgently required. ③ According to body composition analysis (Table 2), the patient was severely malnourished and had abnormally low levels of water, muscle, and fat. ④ Oral intake: patient had normal oral intake where there was no encephalopathy, but it decreased by more than 80% at the onset of encephalopathy.

**Table 1:** The results of laboratory examinations.

Indicators	Admission	Peoperative	Post-1d	Post-5d	Post-11d	Post-1 M	Reference range
WBC (10^9^/L)	5.6	4.1	7.7	2.9	7.5	15.7	3.5-9.5
NEUT (%)	27.7	42.5	82.1	86.2	73.5	71.8	40-70
RBC (10^12^/L)	4.25	2.96	2.96	2.77	2.86	4.26	4.3-5.8
Hb (g/L)	120	93	92	84	90	135	130-175
ALT (U/L)	100.6	22.8	404.1	109.2	22.3	29.0	5-40
AST (U/L)	110.5	31.5	517.7	42.3	15.6	20.2	8-40
ALP (U/L)	219.3	116.1	48.5	47.9	71.5	62.2	47-185
GGT (U/L)	314.5	89.5	68.2	45.9	46.9	48.9	11-50
TBIL (μmol/L)	13.9	3.8	92.1	51.1	90.1	23.8	5-20.5
DBIL (μmol/L)	7.1	2.3	47.0	34.1	57.4	17.2	1.7-6.8
ALB (g/L)	31.9	29.0	33.1	34.6	40.8	40.8	40-55
Triglyceride(mmol/)	2.28	5.17	0.25	0.83	0.59	0.87	0.56-1.7
Cholesterol(mmol/L)	2.87	2.76	1.14	1.74	0.18	3.01	2.9-5.72
AFP (ng/ml)	12.80	6.90	–	–	–	–	0-10
Serum ammonia (μmol/L)	289.0	69.0	28.0	20.0	9.0	9.0	9-30
Uric acid (μmol/L)	433	353	343	304	129	350	90-420
Cre (μmol/L)	45	43	67	60	30	43	44-106
Urea (mmol/L)	2.9	6.3	7.9	10.1	3.2	8.4	2.9-7.5
eGFR	216.5	228.2	136.8	155.4	345.7	228.2	>90

Post-1d, 1 day post-operation; Post-5d, 5 days post-operation; Post-11d, 11 days post-operation; Post-1 M, 1 month post-operation; WBC, White blood cell; NEUT, Neutrophil; RBC, Red blood cell; Hb, Hemoglobin; ALT, Alanine aminotransferase; AST, Aspartate aminotransferase; ALP, Alkaline phosphatase; GGT, Gamma-glutamyl transferase; TBIL, Total bilirubin; DBIL, Direct bilirubin; ALB, Albumin; AFP, Alpha-fetoprotein; Cre, Creatinine.

**Table 2:** Body composition analysis.

	BW(kg)	BMI (kg/m^2^)	Water (e)	skeletal muscle(kg)	Fat(kg)	BMR(kcal)
Admission	33.2	12.3	20.6	14.2	5.2	919 ~ 1041
Post-10 M	46.8	17.4	26.8	19.4	10.4	1143 ~ 1315

BW, Body weight; BMI, Body mass index; BMR, Basal metabolic rate; Post-10 M, 10 months post-operation.

Considering the patient was Child-Pugh class B, and his poor nutritional condition, elevated serum ammonia levels and triglycerides, the clinical pharmacist advised the patient to take Enteral Nutritional Suspension (TPF-D) (ABBOTT LABORATORIES B.V., Netherlands) with vegetable protein orally (500 mL, once a day) in combination with fat-free parenteral nutrition solution (420Kcal) for initial nutritional support. The next day, the patient's serum ammonia was retested and the results revealed a rise from the earlier level (164 μmol/L → 208 μmol/L). After reviewing the literature, the clinical pharmacist suggested that the Enteral Nutritional Suspension (TPF-D) should be adjusted to Enteral Nutritional Suspension (TP-MCT) (Nutricia, Netherlands) (500 mL, once a day), which is rich in medium-chain triglycerides (MCT), in combination with parenteral nutrition (PN) containing structural fat emulsion (Fresenius Kabi AB, Switzerland) (870 Kcal). Serum ammonia steadily improved.

**Figure 1:**
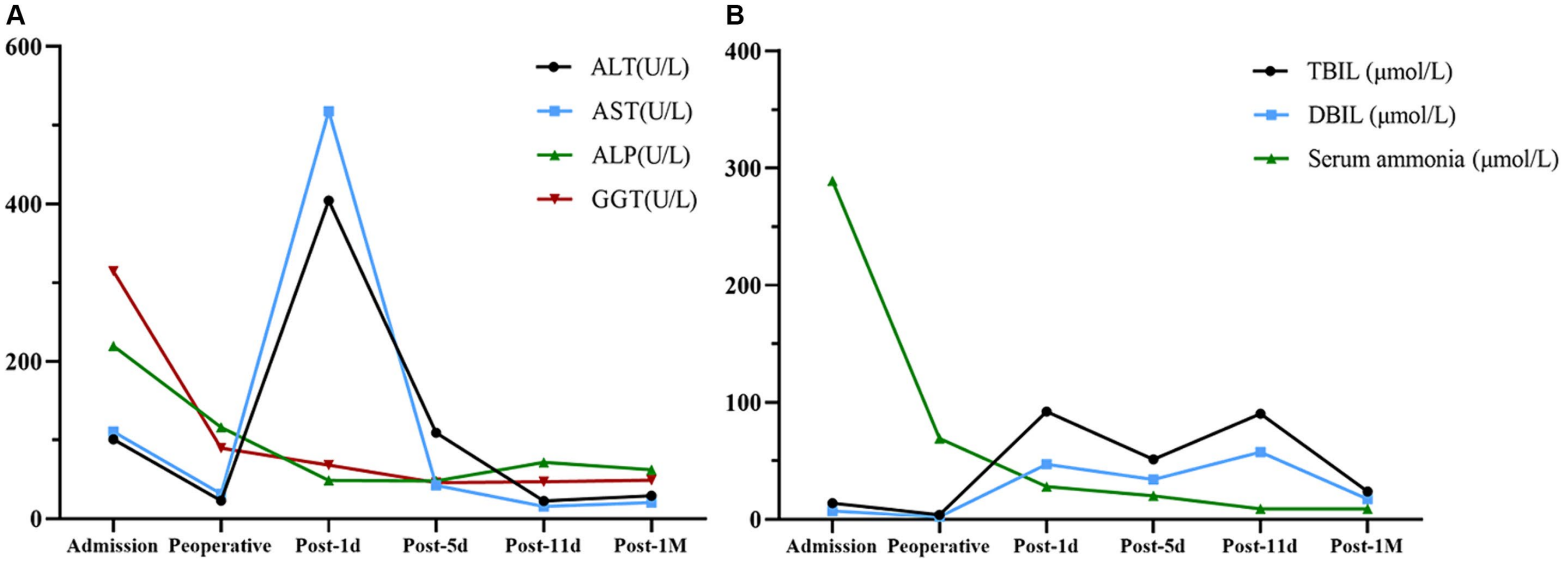
Changes in parameters related to liver function. **(A)** Alanine aminotransferase (ALT), aspartate aminotransferase (AST), alkaline phosphatase (ALP), and gamma-glutamyl transferase (GGT) fluctuation. **(B)** Total bilirubin (TBIL), direct bilirubin (DBIL), and serum ammonia fluctuation.

**Figure 2:**
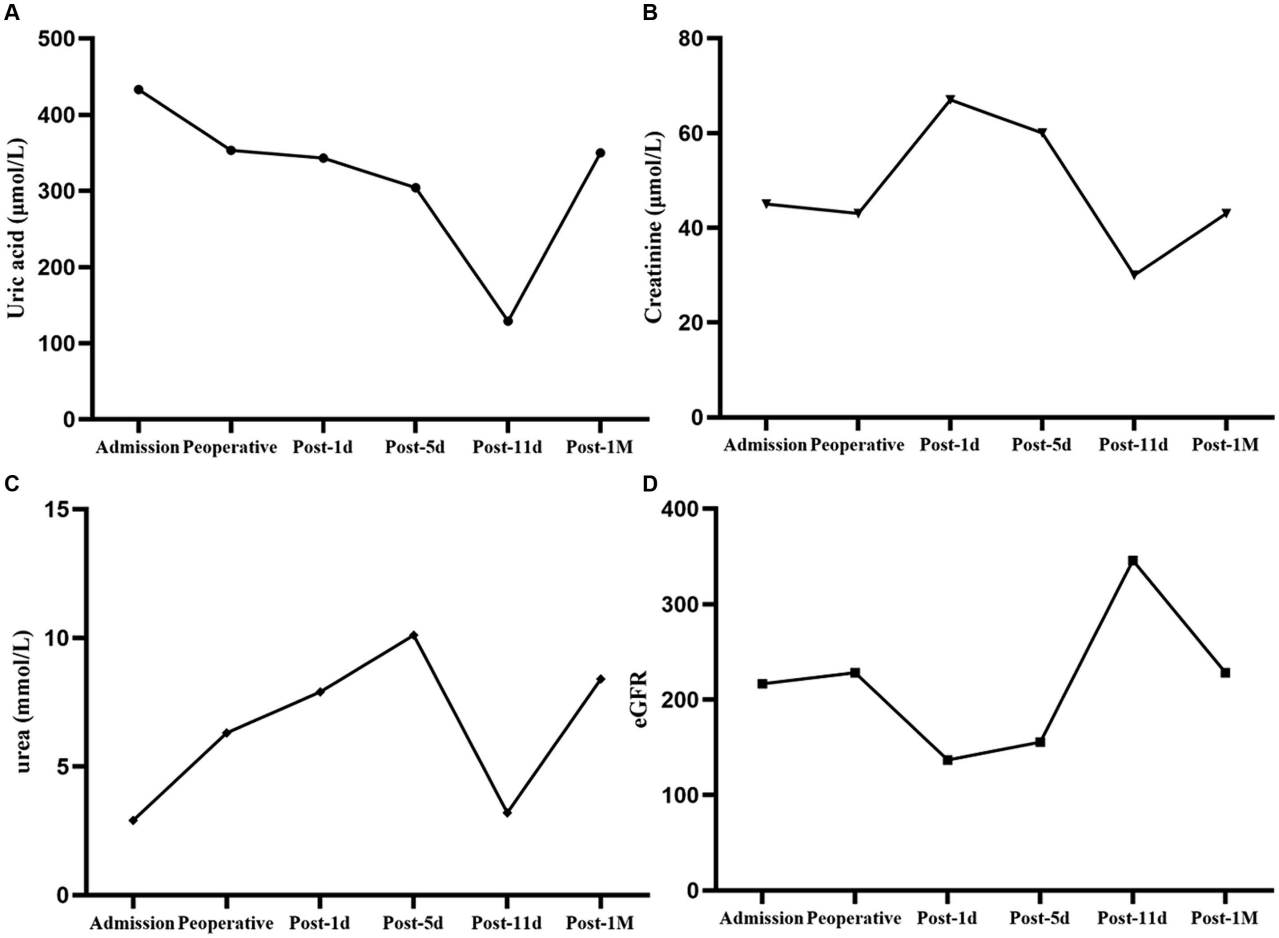
Changes in parameters related to renal function. **(A)** Uric acid fluctuation. **(B)** Creatinine fluctuation. **(C)** Urea fluctuation. **(D)** eGFR fluctuation.

After receiving 7 days of nutritional support, the patient underwent split liver transplantation (left lobe). 24 h post operatively, patient was started on liquid diet and was treated with Enteral Nutritional Suspension (TP-MCT) (500 mL, once a day) in combination with parenteral nutrition containing structural fat emulsion (870 Kcal). Ammonia returned to normal by post operative day 5 and patient refused to take enteral nutritional supplements. Following nutritional assessment, the clinical pharmacist recommended adding a lipid-restricted and protein-rich diet instead of EN combined with PN and formulated individualized dietary recommendations for the patient (Table 3). On postoperative day 11, his oral intake was almost 900 kcal per day, meeting more than 60% of the daily energy needs. The clinical pharmacist considered adding oral Enteral Nutritional Powder (TP) (ABBOTTLABORATORIES.B.V., Netherlands) at 50 g per day while stopping PN for the patient. On postoperative Day 32, the patient's weight increased by 18.7% to 38 kg, and his albumin level improved by 22% to 39 g/L. Because of his good nutritional recovery, patient was discharged home on post operative day 32. The fluctuation of parameters in relation to liver function and renal function during the patient's hospitalization were shown in Figures 1, 2.

**Table 3:** Individualized suggestions for diet.

Date	Actual intake (kcal/d)	Target intake (kcal/d)	Starch (g)	Meat (g)	Egg (g)	Milk (g)	Vegetables (g)
Post-5d	250	1,274	175	125	50	160	500
Post-11d	990	1,274	175	125	50	160	500
Post-10 M	2400	1,504	225	225	50	320	500

Post-5d, 5 days post-operation; Post-11d, 11 days post-operation; Post-10 M, 10 months post-operation.

**Figure 3:**
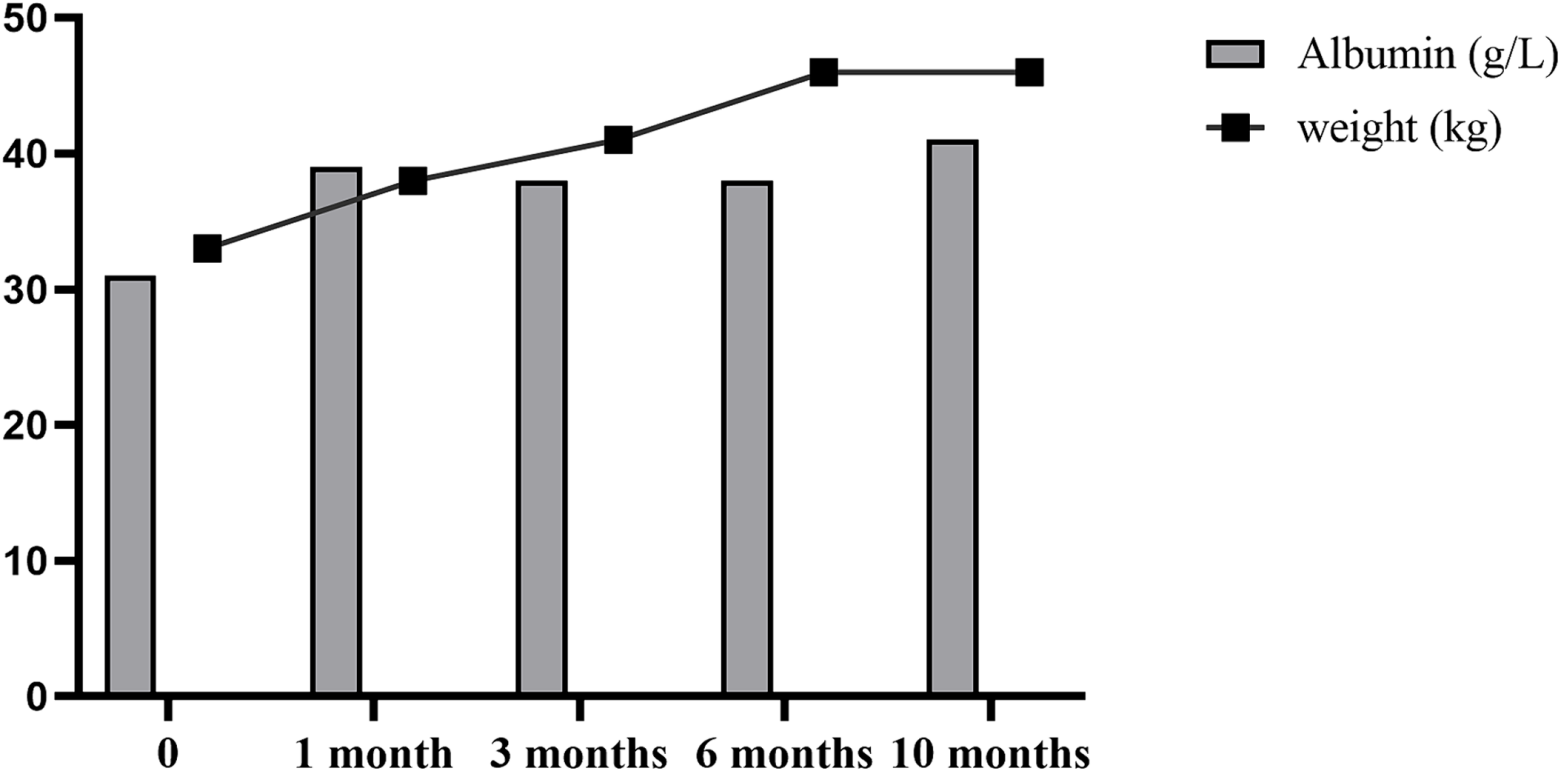
Changes in weight and albumin at 1, 3, 6, and 10 months after surgery.

The patient was followed up at 3, 6, and 10 months after liver transplantation, and his body weight and albumin level all gradually increased (Figure 3). The body composition analysis revealed a 40.96% rise in weight, a 37.14% increase in muscle, and a 100% increase in body fat compared to the preoperative period, which was repeated at 10 months post operation (Table 2). Considering the patient's rapid growth in body fat, the clinical pharmacist advised the patient to adjust his diet to increase the proportion of protein intake, and also provided specific recommendations on the amount of food to eat (Table 3). During 10 months of follow up after liver transplantation, he remained in good condition without an episode of hepatic encephalopathy, required biliary stent placement for biliary stricture, and his liver function remained normal thereafter. Furthermore, he has also maintained a stable weight of approximately 45 kg.

## 3 Discussion

CTLN2 is an autosomal recessive disease caused by mutations in the *SLC25A13* gene which encodes Citrin, the liver-specific isoform 2 of the mitochondrial aspartate/glutamate carrier. The prevalence of citrin deficiency (CD) has been reported to be approximately 1 in 7100 persons, and relatively common in East Asia and Southeast Asia. CD patients demonstrate some typical clinical features with age. Neonatal intrahepatic cholestasis (NICCD) caused by citrin deficiency, which manifests as cholestasis, galactosemia, hypoproteinemia, and dysplasia. After recovering from NICCD, many patients are asymptomatic except for their dietary preferences, while others complain of fatigue, hypoglycemia, dyslipidemia and short stature, which are designated as failure to thrive and dyslipidemia caused by CD (FTTDCD). In adulthood, <10 % CD patients develop CTLN2.

CTLN2 shows clinical manifestations with hyperammonemia and citrullinemia, and repeatedly impaired consciousness. It is reported that the prognosis of CTLN2 patients exhibiting hyperammonemia and serious encephalopathy is poor (9). A review of CTLN2 revealed that while conservative therapies, such as arginine and sodium pyruvate administration, high doses of glycerol and fructose infusion for brain edema, hyperalimentation, a low-protein and high-carbohydrate diet, have provided temporary relief from hyperammonemia, they have not improved the long-term prognosis (3). Several studies have demonstrated that the majority of surviving CTLN2 patients undergoing conservative therapies have been managed with the administration of arginine in conjunction with a carbohydrate-restricted diet. Conversely, there is a risk of mortality associated with inadequate treatments, such as solely relying on intravenous infusion of hyperalimentation fluid containing branched-chain amino acids, implementing a low-protein diet, or using glycerol and fructose infusion for brain edema (10–12). Therefore, if therapies aimed at reducing ammonia levels and providing neuroprotection proved ineffective, the decision for liver transplantation should be made. In this case, the patient also underwent an extended period of conservative therapies, including administration of arginine and ornithine/aspartate to reduce serum ammonia, lactulose for promoting the excretion of intestinal ammonia, diammonium glycyrrhizinate for hepatocyte protection, and ursodeoxycholic acid for jaundice. Despite these interventions, the treatment was ineffective, resulting in the development of HE and necessitating a liver transplantation.

2020 European Society of Parenteral and Enteral Nutrition (ESPEN) practical guideline: Clinical nutrition in liver disease recommends that liver cirrhosis patients scheduled for elective surgery or listed for transplantation should be screened and assessed for malnutrition timely to treat malnutrition before surgery and thereby improve body protein status (4). EN should be used when patients cannot meet their caloric requirements through normal food and/or oral nutritional supplement (ONS), and PN shall be commenced immediately in moderately or severely malnourished patients who cannot be nourished sufficiently by oral and/or enteral route (13). For the intake of nitrogen sources in HE, it is advised to consume nitrogen sources rich in plant proteins and to minimize the intake of animal proteins as much as possible (8, 14). In this case, considering the patient's HE and elevated triglyceride (TG) levels, the initial nutritional support provided before liver transplantation consisted of an enteral nutritional suspension containing vegetable proteins along with a fat-free parenteral nutrition solution. Nevertheless, the patient's serum ammonia levels were reassessed after just 1 day on this nutritional support, and surprisingly, the outcome revealed an increase in serum ammonia rather than a decrease. The nutritional recommendations based on the current guidelines were not found to be applicable for practical management of our patient, we had to formulate a more individualized nutritional treatment plan based on the patient's pathophysiological characteristics and thorough review of relevant literature.In light of this, extensive literature review revealed that medium-chain triglycerides (MCT) have shown promise in ameliorating the symptoms of patients with CD. The use of MCT and lactose-restricted formula has been identified as an effective therapy for intrahepatic cholestasis in NICCD (9). Meanwhile, a low-protein, high-sugar diet, a common treatment for hepatic encephalopathy, exacerbates hyperammonemia in CTLN2. NICCD and CTLN2 share a common cause in CD. Citrin is closely linked to hepatic energy metabolism, and its deficiency is anticipated to impair glycolysis and *de novo* lipogenesis, leading to a persistent and cumulative energy deficit in hepatocytes. MCT undergo rapid hydrolysis, traverse the liver via the portal vein, and are metabolized into acetyl-CoA by β-oxidation, thereby enhancing tricarboxylic acid (TCA) cycle activity, augmenting adenosine triphosphate (ATP) production, and facilitating lipogenesis. Moreover, the heightened ATP levels additionally bolster the efficiency of the ammonia-detoxification system (15). Therefore, in this case, the patient's nutritional support was modified to include MCT-rich enteral nutrition suspension combined with parenteral nutrition featuring a structured fat emulsion. Following this nutritional plan, the patient's serum ammonia levels remained stable, and he underwent liver transplantation without experiencing HE. It was documented that intake of low-carbohydrate helps patients with CTLN2 reduce the accumulation of carbohydrates, and a high-MCT diet ameliorates their disorders of energy metabolism disorders, as well as improving their ammonia-detoxification system. The studies by Hayasaka et al. also confirmed this viewpoint (1, 16). At the same time, CTLN2 patients exhibit a dietary preference to a high-fat diet and distaste for carbohydrates due to their unique metabolic patterns. Nutritionists or nutritional pharmacists need to maintain their dietary habits rich in fat and protein to offset their abnormal metabolism (9).

Restoring intestinal function promptly after surgery is a crucial therapeutic objective of postoperative nutritional support for liver transplantation. ESPEN and EASL both recommend initiating EN as soon as possible after liver transplantation to ensure adequate protein and energy supply, reduce infection rates due to altered intestinal flora, and that PN should be used when EN intake falls below 60% of the target energy and protein requirements. During the perioperative period in liver transplant patients, it is crucial to highlight that the optimal daily energy intake should be 30-35 kcal/(kg d) or 1.3 times the Basal Metabolic Rate (BMR), and protein intake should range between 1.2-1.5 g/(kg d). Research has shown that when EN is administered alone, patients with severe malnutrition have a poor intestinal absorption and tolerance, leading to a high rate of discontinuation EN or unsatisfied intake. In contrast, the combination of EN with PN has been found to be more beneficial for these patients than EN alone (17). In this case, the patient received EN in conjunction with PN 48 h postoperatively to promote early oral feeding. Moreover, we routinely performed nutritional assessments and tailored personalized nutritional support for the patient postoperatively. We also advised dietary interventions to maintain the appropriate protein-fat-carbohydrate ratio. For instance, a recommended ratio of 15-25% protein, 40-50% fat, and 30-40% carbohydrate was suggested (9). Additionally, the patient was required to take long-term immunosuppressants like tacrolimus after surgery to combat immune rejection. Therefore, it is advisable to avoid foods that interact with tacrolimus, such as grapefruit and oranges.

Most liver transplant patients undergo rapid weight gain within 2 years post-surgery attributed to prolonged supplementation, extended period of rest, enhanced liver function, and administration of immunosuppressive medications. Research indicated that up to 26% of liver transplant patients may develop new-onset obesity (18). The increase in weight and obesity can escalate the risk of metabolic syndrome, consequently heightening the chances of cardiovascular morbidity and mortality. The accelerated accumulation of body fat observed in this patient 10 months post-surgery was closely associated with dietary patterns and physical activity levels, potentially escalating the susceptibility to metabolic disorders. Therefore, prolonged dietary management post-liver transplantation should prioritize weight control, particularly focused on curtailing body fat gain, to mitigate the onset of obesity or sarcopenia (19).

## 4 Conclusion

This paper summarizes the nutritional support treatment for liver transplantation in an adult-onset type II citrullinemia (CTLN2) patient as follows: 1. Preoperative severe malnutrition is prevalent among CTLN2 patients undergoing liver transplantation. It is suggested that adhering to the principles of low-carbohydrate, high-medium-chain triglyceride (MCT) nutritional formulas is beneficial in enhancing the nutritional status of CTLN2 patients. 2. For severely malnourished liver transplant patients, EN combined with supplementary PN can even better achieve the target energy requirement. 3. Long-term nutritional management post-liver transplantation should emphasize weight control to mitigate the onset of metabolic disorders.

## Data availability statement

The original contributions presented in the study are included in the article/supplementary material, further inquiries can be directed to the corresponding authors.

## Ethics statement

The studies involving humans were approved by Ethics Committee of Nanjing Drum Tower Hospital. The studies were conducted in accordance with the local legislation and institutional requirements. The participants provided their written informed consent to participate in this study. Written informed consent was obtained from the individual(s) for the publication of any potentially identifiable images or data included in this article.

## Author contributions

YD: Conceptualization, Data curation, Investigation, Methodology, Writing – original draft. Y-YF: Conceptualization, Data curation, Writing – original draft, Project administration. YY: Formal analysis, Supervision, Validation, Writing – review & editing. BH: Formal analysis, Supervision, Validation, Writing – review & editing. W-JZ: Supervision, Validation, Writing – review & editing. D-CY: Supervision, Validation, Writing – review & editing, Funding acquisition, Visualization, Resources. X-JB: Funding acquisition, Investigation, Methodology, Supervision, Validation, Visualization, Writing – review & editing, Resources.

## References

[1] Hayasaka K Numakura C. Adult-onset type II citrullinemia: current insights and therapy. Appl Clin Genet. (2018) 11:163–70. doi: 10.2147/TACG.S162084, PMID: 30588060 PMC6296197

[2] Saheki T Kobayashi K. Mitochondrial aspartate glutamate carrier (citrin) deficiency as the cause of adult-onset type II citrullinemia (CTLN2) and idiopathic neonatal hepatitis (NICCD). J Hum Genet. (2002) 47:333–41. doi: 10.1007/s100380200046, PMID: 12111366

[3] Kimura N Kubo N Narumi S Toyoki Y Ishido K Kudo D. Liver transplantation versus conservative treatment for adult-onset type II citrullinemia: our experience and a review of the literature. Transplant Proc. (2013) 45:3432–7. doi: 10.1016/j.transproceed.2013.06.016, PMID: 24182831

[4] Bischoff SC Bernal W Dasarathy S Merli M Plank LD Schütz T. ESPEN practical guideline: clinical nutrition in liver disease. Clin Nutr. (2020) 39:3533–62. doi: 10.1016/j.clnu.2020.09.001, PMID: 33213977

[5] European Association for the Study of the Liver. EASL clinical practice guidelines on nutrition in chronic liver disease. J Hepatol. (2019) 70:172–93. doi: 10.1016/j.jhep.2018.06.024, PMID: 30144956 PMC6657019

[6] Plauth M Bernal W Dasarathy S Merli M Plank LD Schütz T. ESPEN guideline on clinical nutrition in liver disease. Clin Nutr. (2019) 38:485–521. doi: 10.1016/j.clnu.2018.12.022, PMID: 30712783 PMC6686849

[7] Chinese Society of Hepatology Chinese Society of Gastroenterology. Clinical guidelines on nutrition in end-stage liver disease. J Clin Hepatol. (2019) 35:1222–30. doi: 10.3969/j.issn.1672-5069.2019.05.005 PMC12770166 31177656

[8] Beijing medical association, professional Committee of the Society of parenteral enteral nutrition, expert committee on parenteral enteral nutrition support and dietary interventions for patients with chronic liver disease. Expert consensus on parenteral enteral nutrition support and dietary interventions for patients with chronic liver disease. Chin J Clin Nutr. (2017) 25:1–11. doi: 10.3760/cma.j.issn.1007-8118.2017.02.001

[9] Komatsu M Tanaka N Kimura T Yazaki M. Citrin deficiency: clinical and nutritional features. Nutrients. (2023) 15. doi: 10.3390/nu15102284, PMID: 37242166 PMC10224054

[10] Imamura Y Kobayashi K Shibatou T Aburada S Tahara K Kubozono O. Effectiveness of carbohydrate-restricted diet and arginine granules therapy for adult-onset type II citrullinemia: a case report of siblings showing homozygous SLC25A13 mutation with and without the disease. Hepatol Res. (2003) 26:68–72. doi: 10.1016/s1386-6346(02)00331-5, PMID: 12787807

[11] Yazaki M Takei YI Kobayashi K Saheki T Ikeda SI. Risk of worsened encephalopathy after intravenous glycerol therapy in patients with adult-onset type II citrullinemia (CTLN2). Intern Med. (2005) 44:188–95. doi: 10.2169/internalmedicine.44.188, PMID: 15805705

[12] Tazawa K Shimojima Y Okano T Yazaki M Takei YI Shimojo H. An autopsy case with adult onset type II citrullinemia showing myelopathy. J Neurol Sci. (2007) 253:77–80. doi: 10.1016/j.jns.2006.11.014, PMID: 17196992

[13] Weimann A Braga M Carli F Higashiguchi T Hübner M Klek S. ESPEN practical guideline: clinical nutrition in surgery. Clin Nutr. (2021) 40:4745–61. doi: 10.1016/j.clnu.2021.03.031, PMID: 34242915

[14] Amodio P Bemeur C Butterworth R Cordoba J Kato A Montagnese S. The nutritional management of hepatic encephalopathy in patients with cirrhosis: international society for hepatic encephalopathy and nitrogen metabolism consensus. Hepatology. (2013) 58:325–36. doi: 10.1002/hep.26370 23471642

[15] Bach AC Babayan VK. Medium-chain triglycerides: an update. Am J Clin Nutr. (1982) 36:950–62. doi: 10.1093/ajcn/36.5.950 6814231

[16] Hayasaka K Numakura C Yamakawa M Mitsui T Watanabe H Haga H. Medium-chain triglycerides supplement therapy with a low-carbohydrate formula can supply energy and enhance ammonia detoxification in the hepatocytes of patients with adult-onset type II citrullinemia. J Inherit Metab Dis. (2018) 41:777–84. doi: 10.1007/s10545-018-0176-1, PMID: 29651749

[17] Ding LM Li XC Luo WF Huang XM Xu ZD. Effect of different perioperative nutritional modalities on early postoperative infection after liver transplantation. Chin J Transpl. (2017) 11:211–5. doi: 10.3877/cma.J.issn.1674-2523903.2017.04.004

[18] Brandman D. Obesity management of liver transplant waitlist candidates and recipients. Clin Liver Dis. (2021) 25:1–18. doi: 10.1016/j.cld.2020.08.001, PMID: 33978572

[19] Anastácio LR Ferreira SC. Nutrition, dietary intake, and eating behavior after liver transplantation. Curr Opin Clin Nutr Metab Care. (2018) 21:381–7. doi: 10.1097/MCO.0000000000000491, PMID: 29927763

